# College and Hospital Notes

**Published:** 1899-03

**Authors:** 


### COLLEGE AND HOSPITAL NOTES.

The Medico-chirurgical College of Philadelphia announces a special quiz course for the preparation of candidates for the medical corps of the United States Army, Navy and Marine Hospital examinations.

The course began Wednesday, February 1st, and will continue for four months. Sections will be limited to thirty members. The fee has been placed at $100, payable in advance.

Physicians taking this course are also privileged to attend all clinics given in the Medico-chirurgical Hospital and to obtain practical experience in the several departments of the college dispensary service. Hours will not conflict. Not only will the professional qualifications of applicants be looked after, but experienced teachers will give special attention to history, literature, geography, grammar, mathematics, Latin, physics and all collateral branches.

No lectures will be given, but lessons assigned and thoroughly quizzed over, experience having demonstrated the superiority of this method of teaching.

Candidates for the Army must be between 21 and 29 years of age; Navy and marine service, 21-30.

No extra charges will be made except for material used in the courses on practical anatomy and operative surgery. Instruments will be furnished in the surgical course and every operation in established use performed by members of the class.

The course will be conducted by the following-named teachers; Military surgery, Dr. D. C. Peyton, major and brigade surgeon U. S. army. Hygiene—special attention to food, water, camp and ship sanitation, etc., Seneca Egbert, M. D. Anatomy, Joseph D. Wallace, M. D. Surgery, J. Thompson Schell, M. D. Operative surgery and bandaging, W. V. Laws, M. D. Surgical pathology and Bacteriology, W. Hershey Thomas, M. D. Practice of medicine—tropical diseases included—Albert E. Blackburn, M. D. Physical diagnosis, obstetrics and gynecology, L. Napoleon Boston, M. D. Physics, chemistry, physiology and physiological chemistry, Matthew Beardwood, Jr., M. D. History, Literature, Latin, Grammar and Geography, Clarence Stanley McIntire, Ph. D. Arithmetic, Algebra and Geometry, Arthur B. Turner, A. B.

Those desiring information relating to the course should apply to Seneca Egbert, M. D., Dean of the medical faculty, Seventeenth and Cherry streets, Philadelphia, Pa.

---

THE Brooks Memorial Hospital, Dunkirk, N. Y., has recently held a meeting of its board of directors, which is composed of physicians residing at Dunkirk and Fredonia. They serve as active and consulting surgeons. The officers of the staff in immediate charge of the hospital are, president, Dr. George E. Blackham; vice-president, Dr. R. T. Rolph; secretary, Dr. Frank S. Jackson.

